# Recognizing Human Daily Activity Using Social Media Sensors and Deep Learning

**DOI:** 10.3390/ijerph16203955

**Published:** 2019-10-17

**Authors:** Junfang Gong, Runjia Li, Hong Yao, Xiaojun Kang, Shengwen Li

**Affiliations:** 1 School of Geography and Information Engineering, China University of Geosciences, Wuhan 430074, China; jfgong@cug.edu.cn; 2 School of Computer Science, China University of Geosciences, Wuhan 430074, China; lirunjia@cug.edu.cn (R.L.); yaohong@cug.edu.cn (H.Y.); xj_kang@126.com (X.K.)

**Keywords:** human activity category recognition, social media, deep learning, long short-term memory network (LSTM), temporal information encoding

## Abstract

The human daily activity category represents individual lifestyle and pattern, such as sports and shopping, which reflect personal habits, lifestyle, and preferences and are of great value for human health and many other application fields. Currently, compared to questionnaires, social media as a sensor provides low-cost and easy-to-access data sources, providing new opportunities for obtaining human daily activity category data. However, there are still some challenges to accurately recognizing posts because existing studies ignore contextual information or word order in posts and remain unsatisfactory for capturing the activity semantics of words. To address this problem, we propose a general model for recognizing the human activity category based on deep learning. This model not only describes how to extract a sequence of higher-level word phrase representations in posts based on the deep learning sequence model but also how to integrate temporal information and external knowledge to capture the activity semantics in posts. Considering that no benchmark dataset is available in such studies, we built a dataset that was used for training and evaluating the model. The experimental results show that the proposed model significantly improves the accuracy of recognizing the human activity category compared with traditional classification methods.

## 1. Introduction

The knowledge about which activities and why people perform them at a certain time can be very valuable for identifying healthy lifestyles [1] and can be used in a wide range of applications. For example, doctors could better build the connection between patients’ habits and lifestyles and their health problems based on people’s daily activity patterns. However, collecting trajectory labeled activity categories by questionnaire or by wearable devices is financially costly and may violate personal privacy.

In this paper, we aim to automatically extract high-level logical activity [2] (e.g., sports, shopping, and entertainment) trajectories through a convenient, low-cost source, where most research studies rely on visual (e.g., cameras) and wearable devices to identify low-level physical activities (e.g., standing, walking, and sitting) [3,4] instead of high-level logical activities. For many applications, such as understanding the human lifestyle and healthy, high-level logical activity categories are more informative. There are currently few related studies, and new methods and data sources both need to be developed to achieve this goal.

Currently, people are considered sensors, producing signals on events they are directly involved in or where they are present [5]. People enjoy sharing with their friends what they are currently doing, anytime and anywhere, on social media platforms [6]. Unprecedented quantities of user-generated data on human activity have been collected through smartphones on social media; these data contain texts and time that reflect extensive knowledge about human activity. Due to their widespread rich information attached to social media posts, these social media data can be of great value in human activity recognition. However, the posts on social media are represented as short texts that are very uncertain. Previous researches employed traditional classification methods [7,8,9], which ignore the contextual information or word order in posts and remain unsatisfactory for capturing the activity semantics of the words; there are still some challenges to accurately recognizing human daily actives in social media.

Considering this problem, we propose a general model for recognizing the human activity category based on a deep learning sequence model. To the best of our knowledge, our proposal is the first to recognize activity from social media based on deep learning [10] theory and is the first to fuse text semantics, external knowledge and temporal information to complete this task. In summary, the contributions of this paper are listed as follows:

(1) We propose a general model for human activity classification based on a deep learning sequence model and a series of solutions to capture the activity semantic by embedding, encoding and fusing text semantics, external knowledge and temporal information for machine learning. The model provides methods on how to extract a sequence of higher-level word phrase representations in posts, as well as how to integrate temporal information and external knowledge in posts.
(2) We build an activity-text dataset that contains activity labels, post texts, and release time called Yelp-20k. The dataset contains 14 categories of activities and 28,150 labeled posts; the dataset utilized in this paper can be downloaded via GitHub. The dataset can be taken as a benchmark dataset to facilitate works on human activity recognition.
(3) We present the proposed model and its variants to classify human activity with accuracies of 81.29, 81.99, 82.93 and 82.42%. While all four models perform similarly well for human activity recognition, combining text semantics and temporal information is more robust.

The rest of the paper is organized as follows. In Section 2, we discuss the state-of-the-art literature on mining human activities from social media data. We then present the proposed model in Section 3. In Section 4, we report on the experiment and the analysis of the results in detail. Finally, we conclude the paper in Section 5.

## 2. Related Work

Referring to human activity recognition, many works aim to identify low-level physical activities (e.g., standing, walking, and sitting). Human physical activity recognition is a promising research field due to its applications in healthcare, athletics, lifestyle monitoring, and computer-human interaction. Usually, it can be implemented from three categories of data, such as video, wearable motion sensor data, and multi-sensors data [11,12,13]. Researchers use traditional classification methods such as SVM (Support Vector Machine) and TF-IDF (Term Frequency–Inverse Document Frequency) models to identify human physical activities and develop a syntactic approach that treats activities as sequential text and uses a grammar syntax such as stochastic context-free grammar to identify physical activity categories [7,8,9,14,15,16]. Recently, deep learning has been adopted by combining spatiotemporal dynamic texture descriptors [11].

In contrast to physical activity, human activity recognition in this paper aims to classify high-level logical activities (e.g., eating, shopping, and entertainment). For high-level logical activities recognition, there are different works in the literature using different types of data. These related studies can be summarized in 3 categories.

The first category of works focuses on activity category prediction. Weerkamp proposed an approach to predict the popular activities that will occur in a future time window, such as tonight, tomorrow, and next week, by using a future time window and keywords related to activities [17]. Their input data and objectives are different than this paper, and methods in those studies cannot be used directly for the task of identifying activities.

The second category is human activity recognition using location information in both continuing and noncontinuous types of trajectories. (1) For continuous trajectories, these studies usually use constrained random fields, support vector machines, or Bayesian to identify travel modes with spatiotemporal features of trajectory data [18,19,20,21]. An algorithm was developed for automatically annotating raw trajectories with the activities performed by analyzing users’ stop points to infer the POI (Point of Interest) that the user has visited [22]. These studies mainly use the space-time position information in the trajectory data for discrimination, and the description information when the trajectory is generated is missing. The category of activity identified is relatively simple. In mixed-use plots such as shopping centers and office buildings, the recognition accuracy is not high. (2) Noncontinuous trajectories, such as mobile phone trajectories, and social media are used to classify human activities [23,24]. Beber designed a matching method to infer activities based on the similarity between the trajectory and the POI category profiles, but only a few records can be treated as continuous trajectories [25]. Hasan classified different activity categories based on the category of visited locations [26]. Description-based approaches represent human activities by describing subevents of the activities and their temporal, spatial, and logical structures, reconstructing individual mobility history from a metropolitan-scale smart card transaction dataset [15,18]. Although these approaches can recognize different categories of human activities, they all need detailed description information and abundant temporal, spatial, and logical information for every activity. However, most of the posts on social media do not have location attributes marked because of the limitation of users’ devices and for privacy reasons.

The third category of related works infers the user activity category from crowdsourced texts. Human activities can be expressed not only by images and videos but also by texts. Zhu, Blanke [2] built a multilabel SVM classifier using tweets manually annotated with activities by a crowd workers’ experiment. They constructed an activity recognition model to classify the dataset into 10 activity classes, with accuracies of 76%. This work is most closely related to ours. However, such logical activity recognition from short text posts based on traditional text classification is difficult because it cannot capture the word contextual information and word semantics.

## 3. Model

A post could be a text document, blog post, tweet, etc. For example, from the post “the pizza is very delicious”, we have a high probability of determining that the person who made this post is eating. Through social media platforms that record such data, we can obtain rich information for activity recognition without any additional instrumentation. Therefore, as long as we establish the connection between posts and activities and then classify these posts, we know what kinds of activities people are performing. Thus, by mapping activities to posts, activity classification is similar to a text classification task. In this paper, our objective is to design a model to improve the accuracy of extracting human activity for posts on social media.

Inspired by a deep learning sequence model that has had considerable success in many natural language tasks, including text classification and voice recognition, we will use deep learning to automatically deduce the classification of an activity and recognize an activity from social media texts and time together.

**Figure 1:**
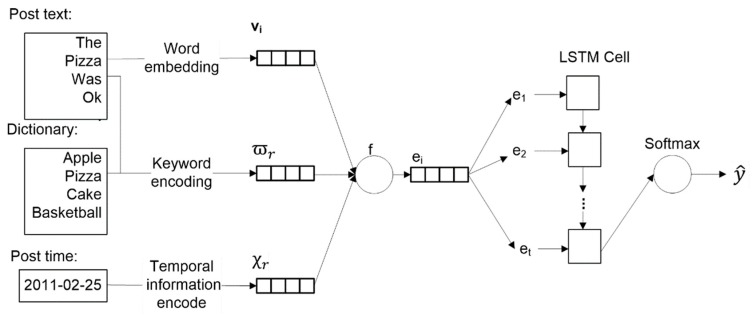
The proposed ALSTM (activity LSTM) model.

The proposed model is derived from a long short-term memory network (LSTM) [27], and we name it ALSTM (activity LSTM). Its architecture is shown in Figure 1 and consists of four main components: word embedding, dictionary knowledge encoding, temporal information encoding, and a long short-term memory network.

In the following subsections, we explain how to recognize human activities from social media with a deep learning sequence model and how to integrate external knowledge and temporal information to improve precogitation accuracy.

We denote a post of T words as $\left\{w^{\left(t\right)}|t=1,\ldots ,T\right\},$ and a corresponding activity category as target y.

### 3.1. Post Word Embedding

Both the word meaning and its context should help us to capture a more precise word characteristic related to human activity. In our model, we use the word embedding model to capture the word meaning of the word and its context in the word sequence.

Word embedding is a model that represents words in sparse spaces as low-dimensional and high-density distributed representations. Compared to traditional representations, such as one-hot representation, which leads to the curse of dimensionality, word embedding is suitable for the neural network input because it embeds rich syntactic and semantic information of the words [28,29]. Therefore, the word embedding model is used to represent the words in posts for the ALSTM input in the paper.

To generate a word embedding vector for each word in posts, the skip-gram model is selected. The skip-gram model is a popular model for building word embedding and is considered state-of-the-art in many NLP tasks [30]. It trains the embeddings of words $w_{1},$ $w_{2},\;\ldots ,\;w_{t}$ in text sequences by maximizing the average log probability:

(1) $$\frac{1}{T}\overset{T}{\underset{t=1}{\sum }}\underset{-c\leq j\leq c,j\neq 0}{\sum }\log p\left(w_{t+j}|w_{t}\right)\;p\left(w_{b}|w_{a}\right)=\frac{\exp\left({\left({v^{\prime }}_{w_{b}}\right)}^{T}v_{w_{a}}\right)}{\sum_{k=1}^{\left|V\right|}\exp\left({\left({v^{\prime }}_{w_{k}}\right)}^{T}v_{w_{a}}\right)}$$

where |V| is the vocabulary from all of the post text, ${v^{\prime }}_{w}$ and $v_{w}$ are two word embedding vectors of word w. Here, $v_{w}$ is the input vector, and ${v^{\prime }}_{w}$ is the output vector.

### 3.2. Dictionary Knowledge Encoding

Generally, posts may contain different keywords that should contribute more than ordinary words for recognizing human activities in social media posts because those keywords have richer activity semantics. The keyword collection is labeled manually into a different category and is called a dictionary. The dictionary can be introduced as external knowledge.

In this paper, we use the TF-IDF model [31] to extract keywords from training data and to generate a vector for each post. The TF-IDF model reflects how important a word is to a single post in the whole corpus. It is often used as a weighting factor. The importance of a word increases proportionally with the appearing frequency in a post but decreases inversely with the appearing frequency in the whole corpus.

The TF-IDF model includes two parts, term frequency and inverse document frequency. The term frequency refers to the number of times that a given word appears in the post. Its mathematical expression can be defined as follows:

(2) $$TF_{w}=\frac{\#w\;appears\;in\;a\;review}{\#word\;in\;a\;review}$$

However, the large TF value of a word cannot represent a post directly because some common words that appear in many posts will have a large TF value. The main idea of inverse document frequency is that if fewer posts that contain word w, the larger the IDF value it has. It can further indicate that word w has a good post differentiation ability. The IDF value can be represented as follows:

(3) $$\mathrm{IDF}=\log\left(\frac{\#posts\;in\;whole\;corpus}{\#post\;containing\;word\;w+1}\right)$$

The reason that the denominator adds 1 is to avoid the denominator being 0. A word that has a high word frequency in a particular post and a low post frequency in the entire corpus can produce a high weight TF-IDF value. Thus, the TF-IDF model can filter out common words and keep important words. The reserved words can express the feature of posts very well. It can be defined as follows:

(4) TFIDF=TF×IDF

We then use the TF-IDF model to calculate the TF-IDF weight value for each word in the posts and select some high-ranking words in different categories as keywords to construct a dictionary. The number of keywords is determined by the user.

The next step is to transform posts into a weight vector based on the dictionary. The transformation process can be defined as follows:

(5) $$\varpi_{r}=\underset{1\leq i\leq m}{\delta }\left(dic\left(i\right)\right)$$

where r is a post, and dic is the dictionary that we mentioned above. δ is the transform function that counts the number of words in the post that appear in different categories of the dictionary, and m is the number of activity categories. After transforming the post by equation (2), we can obtain an m-dimensional weight vector $\varpi_{r}$ of the corresponding post; each element of the vector is the number of words that appear in that category. It should be noted that the size of the dimension of $\varpi_{r}$ is not the same as the size of the dimension of the word embedding vector.

### 3.3. Temporal Information Encoding

Temporal information such as date and week has abundant activity semantics for activity recognition because human activity has a very close relationship to its occurrence time. With the help of temporal information, we can better determine and infer what activities a user produced at a given time.

Therefore, we consider time to be another important feature of a post for classifying the activity category. Some activities only occur during a specific time. We treat temporal information as additional knowledge and integrate it with the post text by embedding temporal information.

We define the date when the post was published as $d_{r}$. According to the data, we can infer the activity that occurs by the day of the week. It can be represented as follows:

(6) $$\chi_{r}=\mu \left(d_{r}\right)$$

where r is a post and function μ can transform date to the day of the week. After processing by the transform function μ, a day of the week is represented as a one-hot vector $\chi_{r}$.

### 3.4. Long Short-Term Memory Networks

Long short-term memory [27] neural networks are a kind of network architecture. They have a strong capability to process sequential data such as text and have been demonstrated as capable of achieving remarkable performance in sequence data classifying, processing and making predictions [32,33].

In this paper, the posts on social media can be treated as sequence data, and we use the LSTM model to process and classify human activities. The probabilities of the human activity categories are calculated in the model and are considered as the weights when predicting the activity category by each post from social media. The post contains multi-dimensional rich information that need to be reduced to a single attribute item, here it is human activity category.

When we process information at the current moment, the LSTM architecture can preserve and utilize useful information in previous posts but discard useless information. As shown in Figure 1, the vectors generated in the above 3 subsections are combined into a vector by the f function. Here, the *f* function can be defined as:

(7) $$e_{t}=f\left(v_{t},\chi_{r},\varpi_{r}\right)=\left[v_{t};\chi_{r};\varpi_{r}\right]$$

where the semicolons represent row vector concatenation.

At step t, the input is the discrete word $w_{t}$, and the corresponding word embedding is $e_{t}$. The LSTM cell is a repeated unit for each time step as in a standard RNN. We obtain a post target vector $h_{t}$ after training with the LSTM parts. At each time step, the output of the unit is controlled by a set of gates as a function of the old hidden state $h_{t-1}$ and the input at the current time step $e_{t}$, the forget gate $f_{t}$, the input gate $i_{t}$, and the output gate $o_{t}$. The transition functions of three gates are defined as follows:

(8) $$i_{t}=\sigma \left(W_{i}\cdot \left[h_{t-1},e_{t}\right]+b_{i}\right)$$

(9) $$f_{t}=\sigma \left(W_{f}\cdot \left[h_{t-1},e_{t}\right]+b_{f}\right)$$

(10) $$o_{t}=\sigma \left(W_{o}\cdot \left[h_{t-1},e_{t}\right]+b_{o}\right)$$

Here, $W_{i},W_{f}\;and\;W_{o}$ are parameter matrixes for training, σ is the logistic sigmoid function that has an output in [0,1]. As well, the current memory cell $c_{t}$ and hidden state $h_{t}$ can be calculate by:

(11) $$q_{t}=\tanh\left(W_{q}\cdot \left[h_{t-1},e_{t}\right]+b_{q}\right)$$

(12) $$c_{t}=f_{t}⊙c_{t-1}+i_{t}⊙q_{t}$$

(13) $$h_{t}=o_{t}⊙\tanh\left(c_{t}\right)$$

Here, $q_{t}$ denotes new information in current cell, $W_{q}$ is parameter matrix for training, tanh denotes the hyperbolic tangent function that has an output in [−1, 1], and ⊙ denotes the elementwise multiplication.

To understand the mechanism behind the architecture, we can view $f_{t}$ as the function that controls to what extent the information from the old memory cell is going to be thrown away, $i_{t}$ controls how much new information is going to be stored in the current memory cell, and $o_{t}$ controls what to output based on the memory cell $c_{t}$.

For recognizing the human activity category in a post, we regard the output of the hidden state at the last time step of the LSTM cell as the document representation. We connect this target vector to a fully connected layer to classify the activities. We use the Softmax [34] function to calculate the prediction probability $p_{i}$ of a post in each activity category and finally determine which kind of activity it belongs to according to the maximum probability $p_{max}$.

After the activation function, the model takes categorical cross-entropy [35] as a loss function. Its mathematical expression can be derived as follows:

(14) $$L\left(h^{\left(r\right)},y^{\left(r\right)}\right)=\overset{k}{\underset{j=1}{\sum }}1\left\{y^{\left(r\right)}=j\right\}\log\left(\hat{y_{j}^{\left(r\right)}}\right)$$

where $h^{\left(r\right)}$ is the $h_{t}$ vector of post r, $y^{\left(r\right)}$ is the activity category that post r really linked, and $\hat{y_{j}^{\left(r\right)}}$ is the predicted activity category for post r; 1{condition} is an indicator such that 1{condition is true} = 1; otherwise, 1{condition is false} = 0.

## 4. Experiments

In this section, we first describe the built dataset and the experimental parameter settings and evaluate our ALSTM model with traditional classification methods, such as SVM and TF-IDF.

### 4.1. Data

To evaluate the performance of the proposed method, we first built a benchmark dataset from Yelp (www.Yelp.com). Yelp is an online community whose members spontaneously publish posts containing specific public and commercial locations. As of August 2009, “Yelpers” authored over 7 million posts about their personal experiences with restaurants, shopping, beauty, arts, entertainment, and events [36]. Yelp posts are associated with a specific location and are therefore less ambiguous for manually recognizing human activity. Therefore, we choose Yelp as our data source and manually label every post into different human activity categories. Note that the built dataset does not contain position information and is used to infer the human activity category mainly by post text and post time because most posts on social media do not reference geolocation. Example of user posts and business information are illustrated in Table 1 and Table 2.

To build a dataset for training and testing, posts from collected Yelp data were labeled into 14 categories by considering the post text, post time and referenced businesses information. Random sampling was performed according to the rule of about 2000 posts per category, finally a dataset with 28,150 posts was built. It should be noted that only 863 posts of “listening” are selected because there are no enough posts in this category.

The 14 activity categories, including eating food, beauty and spa, entertainment, and travel, are listed in Table 3. A record in our dataset is described as a quadruple (uid, text, time, label), where uid is the userid, text is what a user posts to social media, label is what activity the user performs. This dataset provides temporal and textural information together to help researchers study human activity with social media. In this paper, the dataset is named Yelp-2k because there are approximately 2000 samples in most categories.

As shown in Table 3, the distribution of samples in each category is relatively balanced, except that the number of samples in the listening category is small due to the limitation of source data.

We should also note that the number of samples in the dataset did not differ significantly during the days of the week, which means that it is impossible to conduct user activity categories based only on the day of the week of the posts.

To construct a dictionary, we then use the TF-IDF model to calculate the TF-IDF weight value for all words in the posts and manually select the top 2735 words as keywords. The number distribution of the selected keywords over the categories is listed in Table 4.

### 4.2. Experiment Settings

We use a random sampling method to extract 70% of the records from Yelp-2k built in the last section for training and the remaining 30% of the records for the test.

To transform a discrete word input into a continuous vector, we use the word2vec model to build the word embedding matrix. The dimension size of the word embedding vector is set to 100.

We also set the dimension size of the LSTM hidden layer to 128, 256, 512 and training 500 epochs. The Adam method is adopted as the optimizer. The dropout parameter is set to 0.5 in the network, and the learning rate is set to 0.001.

Finally, we implemented the network model with TFLearn [37] neural network framework.

### 4.3. Results

By combining the components in Figure 1, four variants of ALSTM can be derived: ALSTM-BASIC is the model composed of the post words embedding component and the LSTM component. ALSTM-DE is the model that includes the post words embedding component, dictionary embedding component and the LSTM component. ALSTM-TE is the model composed of the post words embedding component, temporal information embedding component and the LSTM component. ALSTM-DE-TE is the model composed of all the components in Figure 1.

#### 4.3.1. Overall Accuracy

We evaluate the four ALSTM variants and compare them to traditional classification methods, such as SVM and TF-IDF. The accuracy for each activity category on Yelp-20k is calculated and listed in Table 5.

As shown in Table 5, the proposed 4 ALSTM models achieved significant improvements in the task of recognizing human activities compared to traditional methods such as SVM and TF-IDF. All four ALSTM models achieved the best accuracies in some categories.

Among the four ALSTM models, the ALSTM-TE model achieved the highest accuracy in the whole dataset. It should be noted that the number of samples in each category did not differ significantly during the days of the week, as mentioned in Section 4.1, which should be interpreted as the association of user activity categories between post content and time being a potential association, and a deep learning sequence model has exploited this potential association. Perhaps this is the benefit of deep learning.

Compared to ALSTM, the ALSTM-DE model achieved an improvement because the model considers both word embedding and dictionary encoding.

However, the ALSTM-DE-TE model, which combines word embedding vectors, dictionary encoding vectors, and temporal information encoding vectors together as input, achieved a slightly lower average accuracy compared with the ALSTM-TE model. Its accuracy was still higher than that of the ALSTM-DE model. We argue that there is a certain conflict between the information from the dictionary and time. It should be noted that the ALSTM-DE accuracy is more balanced in all categories in which there is higher accuracy in the worse category than ALSTM-TE.

Finally, a nonnegligible result is that the TF-IDF model performed well in certain categories and achieved the best accuracy in the car-related activities category, which is not surprising because here the TF-IDF model is a classification based on manually labeled keywords, and there are some highly differentiated keywords with car-related activity semantics. This result illustrates the importance of artificial knowledge for recognizing human activity from natural language. Of course, we must also realize that the scope of adaptation of this method is still limited.

In a word, the proposed models achieved a great performance for recognizing human daily activity, because that they can successfully capture both words meaning and the contextual information or word order in posts. As well, both external knowledge and temporal information can contribute to the improvement of accuracy and robustness of our task.

#### 4.3.2. Accuracy and Loss Curve

**Figure 2:**
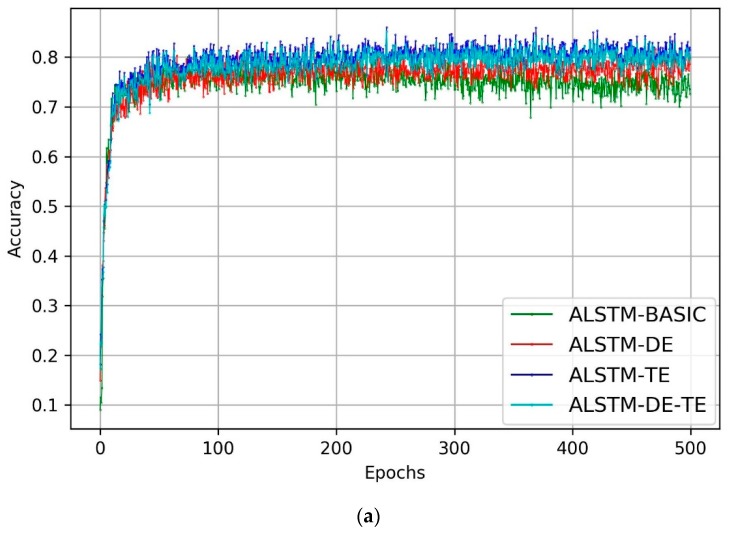

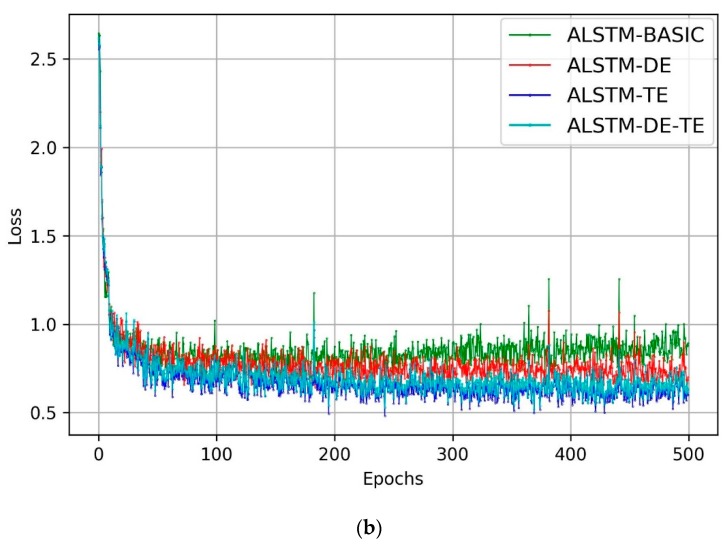
(**a**) Accuracy and (**b**) loss curve.

We calculated the accuracy rate and loss when the models were trained. The accuracy rate and loss curve of the four ALSTM variants are shown in Figure 2. As shown in Figure 2a, the accuracy of all the models increased first, reaching a higher accuracy rate and maintaining relative stability after approximately 80 epochs. From Figure 2b, we can see that the loss of all four models decreased quickly in the first 50 epochs and slightly fluctuated in the last training process. Both Figure 2a,b show that the ALSTM-TE performed best with higher accuracy and lower loss value.

#### 4.3.3. Confusion Analysis Between Categories

In the field of machine learning and the specification of statistical classification, a confusion matrix is a specific table layout that allows visualization of the performance of an algorithm or model, typically in supervised learning [38]. Each column of the matrix represents the instances in a predicted class, while each row represents the instances in an actual class. The confusion matrix can be used to determine whether there is some confusion in different categories.

**Figure 3:**
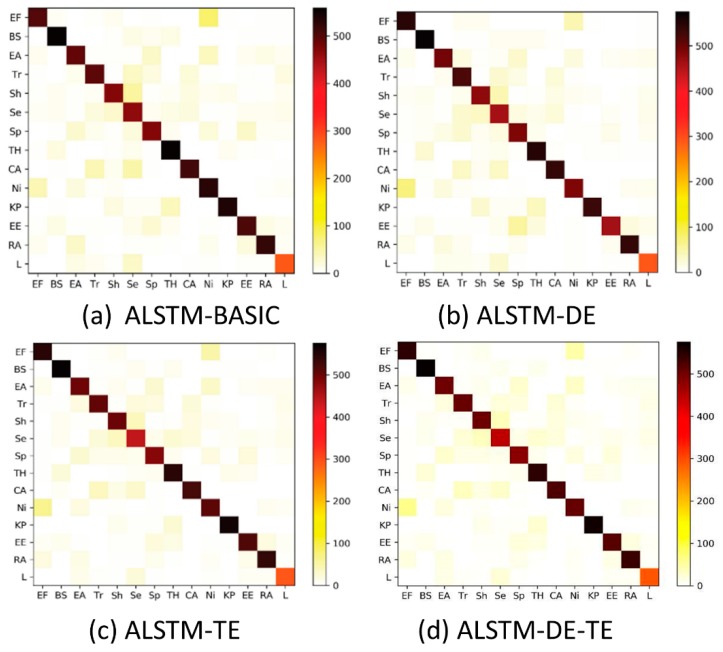
Heat maps of activities for the four models.

We conduct 4 confusion matrixes of the ALSTM series of models in Figure 3, and then we translate them to 4 heat maps to intuitively compare changes and differences. The sum of each heat map column in Figure 3 represents the samples predicted to be in that category by the model.

In addition, the sum of each heat map row represents the actual number of samples in that category. The abbreviations in 4 figures are “EF”, “BS”, “EA”, “Tr”, “Sh”, “Se”, “Sp”, “TH”, “CA”, “Ni”, “KP”, “EE”, “RA” and “L”, which represent the categories “eating food”, “beauty & spa”, “entertainment activity”, “travel”, “shopping”, “services”, “sports”, “treating & health”, “car-related activities”, “nightlife”, “keep pets”, “engaged in education”, “religious activities” and “listening”.

The above 4 heat maps clearly illustrate the accuracy of each category and the distribution of correct and incorrect samples in each category. We also found that the categories of “eating food” and “nightlife” have the maximum confusion in Figure 3, which means that some people eat food so late and eating is one of the common activities of people who are active overnight. Some activities such as “car-related activities” have less confusion with the “nightlife” category in both 4 heat maps, which means that there is a large difference in how to express “eating food” and “car-related activities” in post texts.

Figure 3 also shows that the ALSTM-DE-TE model performed the best for dealing with category confusion, although its overall accuracy was slightly lower than that of the ALSTM-TE model. We believe that the ALSTM-DE-TE model may perform better in other datasets.

#### 4.3.4. An additional Experiment in Selected Category Samples

Considering that most of the people who made posts on comment websites are closely related to daily activities, some statement of posts was not clear enough. We filtered uncommon activities such as “entertainment activity”, “services”, “car-related activities”, “nightlife”, and “listening” and reserved the remaining 9 activities. Then, we calculated the accuracy for each activity category again. We also conducted comparative experiments with our methods and list the results in Table 6.

Compared with Table 5, Table 6 shows that these models achieved better results when the number of categories of activities decreased because fewer categories have less confusion with others. As we expected, all ALSTM models achieved a significant improvement in taking traditional methods as baselines, and the ALSTM-TE model still had the best performance compared with other methods. The gap between those models deceased according to the data in Table 6.

## 5. Conclusions

For accurately recognizing human daily activity in social media posts, we proposed a model based on a deep learning sequence model. The proposed model includes a word embedding component, a dictionary knowledge encoding component, a temporal information encoding component, and an LSTM component. Considering that no datasets are available in such studies, we built a social media benchmark dataset with 14 activity categories and 28,150 labeled posts for training and testing. The proposed models achieved state-of-the-art performance with high accuracies for the recognition of human activity since they successfully extracted user activity information in posts from sequences of higher-level representations while preserving the sequence order of words. In the model, both external knowledge and temporal information can contribute to the improvement of accuracy and robustness of the task.

For future work, user attributes can be fused to infer latent semantics of posts to further improve the classification performance. Additionally, we will introduce more knowledge, such as Wikipedia and Freebase data, into models to improve the accuracy of our model. Due to the limitation of the data source, the time in the built dataset is represented as the date, which could be improved by including new data sources, such as Facebook.

## Figures and Tables

**Table 1:** An example of a post on Yelp.

Attribute	Value	Description
Post id	X7mDliDB3jEiPGPHOmDzyw	Unique id for every post
User id	msQe1u7Z_XuqjGoqhB0J5g	Unique id for every user
Business id	iCQpiavjjPzJ5_3gPD5Ebg	Unique id for every business
Star	2.0	Star rating for the business
Date	2011-02-25	Time when the post was published
Post	The pizza was ok. Not the best I’ve had.	The post that the user published

**Table 2:** An example of business information.

Business Information		Description
Id	iCQpiavjjPzJ5_3gPD5Ebg	Unique id for every business
Name	Minhas Micro Brewery	The name of the business
Address	1314 44 Avenue NE	The address of the business
City	Calgary	City where the business is located
Postal code	T2E 6L6	The postal code for the business
Post count	24	The number of posts the business received
Stars	2.0	Star rating for the business
Categories	Pizza, Restaurants, Food	The category for the business
Hours	Monday: 8:30–17:00, Tuesday: 11:0–21:00, Wednesday: 11:00–21:00 Thursday: 11:00–21:00	Business time for every company

**Table 3:** Activity categories and their distribution in time.

Category	All	Monday	Tuesday	Wednesday	Thursday	Friday	Saturday	Sunday
Eating food	2087	278	299	313	230	299	327	341
Beauty & spa	2265	323	353	295	357	340	308	289
Entertainment	2085	294	293	245	296	284	351	322
Travel	2085	326	327	292	279	279	289	293
Shopping	2085	304	336	272	314	289	265	305
Services	2085	338	379	320	335	239	182	292
Sports	2085	368	298	305	306	245	254	309
Treating & Health	2085	354	378	352	351	214	150	286
Car-related activities	2085	315	328	325	350	275	222	270
Nightlife	2085	317	290	286	253	293	320	326
Keep pets	2085	302	322	326	314	294	241	286
Engaged in education	2085	326	317	346	307	236	249	304
Religious activities	2085	292	282	260	231	239	427	354
Listening	863	142	146	140	127	88	103	117
total	28,150	4279	4348	4077	4050	3614	3688	4094

**Table 4:** Keywords and the number of categories in the dictionary.

Category	Number
Eating food	120
Beauty & spa	49
Entertainment	85
Travel	98
Shopping	135
Services	99
Sports	212
Treating & Health	296
Car-related activities	306
Nightlife	259
Keep pets	258
Engaged in education	326
Religious activities	248
Listening	244

**Table 5:** Overall accuracy.

Activity	Accuracy					
	SVM	TF-IDF	ALSTM-BASIC	ALSTM-DE	ALSTM-TE	ALSTM-DE-TE
Eating food	0.6502	0.4281	0.8019	0.8722	0.8738	0.8706
Beauty & spa	0.6384	0.1040	0.8944	0.9200	0.9200	0.9200
Entertainment activity	0.5376	0.2064	0.7888	0.7936	0.8000	0.8384
Travel	0.5408	0.1504	0.7904	0.8368	0.8112	0.8352
Shopping	0.4480	0.2304	0.7520	0.7632	0.8016	0.7888
Services	0.4432	0.2112	0.7440	0.7360	0.7056	0.7296
Sports	0.4064	0.5200	0.7536	0.7824	0.7712	0.7440
Treating & Health	0.5072	0.8288	0.8912	0.8800	0.8736	0.8496
Car-related activities	0.4384	0.8928	0.8176	0.8608	0.8400	0.8256
Nightlife	0.4544	0.6864	0.8496	0.7792	0.8128	0.7744
Keep pets	0.4624	0.4960	0.8656	0.8512	0.9040	0.8832
Engaged in education	0.4080	0.7808	0.8112	0.7376	0.8288	0.8096
Religious activities	0.5296	0.5856	0.8368	0.8624	0.8592	0.8736
Listening	0.3215	0.5749	0.7629	0.7902	0.7929	0.7766
Overall accuracy	0.4897	0.4753	0.8129	0.8199	0.8293	0.8242

**Table 6:** Accuracy of the selected 9 category activities.

Activity	Accuracy					
	SVM	TFIDF	ALSTM-BASIC	ALSTM-DE	ALSTM-TE	ALSTM-DE-TE
Eating food	0.8067	0.6230	0.9361	0.9281	0.9425	0.9377
Beauty & spa	0.6736	0.1728	0.9248	0.9200	0.9120	0.9472
Travel	0.6720	0.4336	0.8992	0.9168	0.9120	0.8880
Shopping	0.5376	0.4224	0.8384	0.8528	0.8544	0.8800
Sports	0.4752	0.6288	0.8256	0.8096	0.8432	0.8512
Treating & Health	0.5488	0.8864	0.9040	0.9056	0.9328	0.8704
Keep pets	0.4928	0.5440	0.8928	0.9040	0.8944	0.8896
Engaged in education	0.4528	0.8320	0.8288	0.8432	0.8496	0.7792
Religious activities	0.6160	0.6448	0.9056	0.9152	0.9008	0.9184
Overall accuracy	0.5862	0.5764	0.8839	0.8884	0.8935	0.8846
